# Iritis

**Published:** 1917-01

**Authors:** James C. Carter

**Affiliations:** Denison, Texas


### Iritis.

BY JAMES C. CARTER, M. D., DENISON, TEXAS.

Inflammation of the iris is the most frequent affection to which this structure is liable, and always preceded by hyperemia.

*Symptomalology.*—It may arise as a symptomatic affection in the course of syphilis, gout, rheumatism, diabetes, tuberculosis, gonorrhea, etc., or it may occur as the results of an extension of inflammation from adjacent ocular structures.

It is also due to traumatism, exposure to cold and wet, febrile diseases, new growths, etc. Dental caries has also been recorded as a causal factor. The disease is sometimes encountered as an idiopathic affection.

The symptoms and signs of various forms of iritis vary more or less according to the character and intensity of the inflammation, but on the whole are rather constant.

Ciliary injection present in disease of the iris, ciliary body, and cornea, derived from the anterior ciliary vessels, from perforating episcleral branches, most marked immediately around the cornea, fading as the cornea is approached. Color of injection is pink, violacious, or lilac.

Texture, fine; usually not accompanied by abnormal secretions.

Subjective symptoms include neuralgic pain, distributed over the course of the fifth cranial nerve, and worse at night, more or less tenderness on palpatation over ciliary region, photophobia, lacrymation, tiring of accommodation, diminution in the acuity of vision, malaise, fever, and nausea.

According to its course, iritis may be divided into acute, subacute, and chronic. Acute cases require several weeks to complete their course, but recurrences are frequent, and the affection is liable to become chronic.

One or both eyes may be attacked. Usually the disease begins in the second eye when it is subsiding in the first. It may be congenital or acquired.

Simple or plastic iritis is the most frequent form of the affection encountered. The term plastic has been applied on account of synechiae, which generally forms. It is characterized by marked ciliary and pericorneal congestion, slight edema of the subconjunctival tissue, haziness of the aqueous, discoloration of the iris, contraction and immobility of the pupil, posterior synechiae, or adhesions between the iris and the anterior lens capsule.

This type of the disease is also distinguished by the pouring out of an exudate into the pupillary space, forming a false membrane in that area. Occasionally the exudate consolidates and is deposited as a gelatinous mass in the bottom of the anterior chamber. In mild cases, it may be absorbed.

*Etiology.*—In from 30 to 60 per cent of cases this affection can be directly traced to acquired syphilis. Of the remaining cases, a large number are attributed to extension of inflammation of the cornea, sclera, ciliary body, choroid, etc.

Injury, infection, fevers, rheumatism, etc., are also causes. It may occur without obvious causes.

*Course.*—It may be acute, subacute, or chronic.

A case may be acute and run its course within a few weeks with total absorption of the exudate and a complete return to normal. Such cases are rare, however, and the most frequent wind-ups are by adhesions between the iris and the lens capsule. The binding down of the iris throughout the whole extent of its pupillary edge, although the pupil itself remains clear, is denominated exclusion or seclusion of the pupil. If the pupil is filled in with opaque inflammatory deposit, the term occlusion of the pupil is applied. With extension or annular synechiae, the angle of the anterior chamber becomes obliterated, the iris, owing to the exudation behind it, is bulged forward, except around its pupillary margin, which is bound down, so that a crater-like depression is evident and the appearance denominated iris bombe is developed. This leads to increased tension.

Secondary glaucoma and even shrinking of the vitreous detachment of the retina and atrophy of the eyeball, unless the communication between the anterior and posterior chambers of the eye is restored by operative measures.

*Pathology.*—Systemic writers at one time were accustomed to divide iritis into the varieties: Plastic, parychymetous, and serious iritis.

A more accurate classification from a pathologic standpoint is acute, chronic, purulent, and nodular, and of which it would take too long to explain each one of these separately.

*Treatment.*—Of course, the treatment depends upon the form of iritis that you have to contend with, but, in all cases, your atrophine, scopalomin, dionin are always indicated. I have seen some cases wherein the leeches were applied to the temple, where seemingly everything else had failed, and this would give wonderful relief. As a matter of fact, there are a hundred and one things which might be used, but, when it comes down to treating a case intelligently, you have to treat it according to whatever type it is, whether it be specific, rheumatic, or otherwise. Heat, dry, moist are always indicated. But before using any of the midriatics you want to be sure that it is iritis, and not glaucoma, as some disastrous results have been brought about when using atropia solution in a supposed to be iritis when really and truly it was a true case of glaucoma.

Subcontivial injections of cyanide of mercury, in some cases, render great service, and also salvarsan, in specific iritis, and a moist alcoholic dressing. In your rheumatic cases, of course, then your salicylate of sodium, salicylic acid and aspirine are indicated.

---

Dr. W. E. McCaleb, of Manor, Texas, has recently moved to Austin, and is pleasantly located in the Scarbrough building.

---

Leo McDaniel, of Cairo, Ill., has a proposition that will interest every physician who owns an automobile. He takes two old worn out tires and makes a new one for you. Let him tell you how he does it.

---

The Phenadul Chemical Co., of Boston, Mass., feel sure that they possess a superior production in Phenadul powder. It must have merit to win the commendation of such noted pathologists as Professors Wm. T. Councilman and W. F. Whitney, of Harvard Medical School, and Professor Timothy Leary of Tufts Medical School.

